# Journalists’ networks: Homophily and peering over the shoulder of other journalists

**DOI:** 10.1371/journal.pone.0291544

**Published:** 2023-10-18

**Authors:** Qin Li, Hans J. G. Hassell, Robert M. Bond

**Affiliations:** 1 School of Communication, The Ohio State University, Columbus, OH, United States of America; 2 Department of Political Science, Florida State University, Tallahassee, FL, United States of America; University of Vienna: Universitat Wien, AUSTRIA

## Abstract

Social media plays an important role in how journalists gather and report news. To understand journalists’ professional environment, we examine the networks of journalists on Twitter who cover politics for U.S. newspapers in conjunction with a sample of journalists who completed a survey. By combining both their network data and survey responses, we examine the distribution of journalists’ ideology (n = 264) and journalistic values (n = 247); and using the network data, we examine the directional relationships between journalists working at large and small papers (n = 4,661). We find that journalists tend to form connections with those who share similar journalistic values. However, we find little evidence that journalists build professional relationships based on similarity in political ideology. Lastly, journalists at larger media outlets are more likely to be central in journalists’ Twitter networks, providing evidence that journalists look to other journalists at larger outlets for direction in news coverage. Our evidence provides unique insights into how social media illuminates journalists’ professional environment and how that environment may shape news coverage.

## Introduction

“*News is the result of the methods newsworkers employ*.”

—Mark Fishman [1].

The daily routines of journalists shape their perceptions of what is newsworthy and how to cover the news [2–4]. Social media, especially Twitter, is an integral and essential part of journalists’ daily professional routines, information gathering methods, and perceptions of news relevance [5–10]. Social media enhances traditional news making processes, provides perspectives on professional trends in journalism, and allows efficient access to sources and news ideas [11].

Social media, specifically Twitter, has affected the professional environment in which journalists work and as well as news gathering processes and news judgments [9, 10]. Many journalists perceive stories gathered from Twitter of equal or greater value than those gathered using traditional methods [12]. The shape and diversity of journalists’ Twitter networks are important because of the impact they have on professional norms [13, 14], the potential for the creation of an ideological echo chamber for reporters [9], and ultimately on perceptions of what is newsworthy and how to cover it [15, 16].

Yet, despite the importance of social media networks for news production, research on journalists’ use of social media has primarily focused on the content that journalists share [5–8, 17] or how readers react to journalists’ social media content [18–20]. To our knowledge, few studies have examined the structure and diversity of journalists’ networks [21, 22]. Moreover, the analysis of journalists’ engagement with each other on social media has primarily focused on gender, outlet, beat, or geographic location [23] rather than ideology, journalistic values or whether journalists “peer over the shoulders” of their colleagues at larger outlets.

To this end, we examine the Twitter network of roughly 5,000 U.S. political newspaper journalists combined with a large-scale survey of those same journalists and data on their employment. Using this data, we examine the nature of the professional and social environment in which journalists work. Drawing on both journalism research and social network concepts and methods, we test whether journalists have professional relationships with other journalists who hold similar professional values and personal political beliefs. In addition, while other work has examined individual journalists’ decisions to “peer over the shoulder” of other journalists [15], we examine the network of journalists more holistically to test whether journalists working for larger newspapers are disproportionately more central to these networks.

This study makes the following contributions to understanding journalists’ professional environment. First, by examining a large-scale Twitter network of journalists who cover politics for U.S. newspapers we provide quantitative evidence that speaks to long-standing media and journalism theories [15, 24, 25]. Specifically, we document the level to which journalists “peer over the shoulder(s)” [25] of those who work at larger papers. Combining Twitter data with survey data, we document the relationship between journalists’ professional connections and similarity in the journalistic values they hold. Above and beyond homopohily based on demographic characteristics, we examine *value homophily* (i.e., homophily based on shared values, beliefs, and attitudes) [26], which reveals a nuanced picture of the role played by homophily in journalists’ professional networks. In addition, we use a whole-network perspective to study journalists’ networks. Relying on the network concept of betweenness centrality [27], we examine network position’s importance in information diffusion, and explain the relationship between network positions they hold and journalists’ individual-level characteristics.

## Creating the news through journalists’ connections

Journalists are prominent users of Twitter, and many news outlets encourage or require journalists to have an active presence on the platform [5–8]. While previous work on journalists and social media has primarily focused on content production [5–8, 17] or readers’ reactions to that content [18–20], we focus instead on the structure and diversity of the Twitter networks of journalists, illuminating the professional and news production environment that journalists inhabit. Although we know a great deal about where citizens go for news, we know much less about the social and the professional environment in which journalists work. Despite limited knowledge about journalists’ online professional networks, this professional environment plays an important role in shaping perceptions of what is newsworthy and how news should be covered [2–4, 9, 13].

Using the Twitter networks of journalists enables us to test important theories of news production. Journalists regularly communicate with other journalists and take cues from each other in creating expectations governing journalistic behavior about what is newsworthy [3, 28, 29]. As journalists process this information, they come to understand the norms of their profession, what is appropriate to cover, and how topics should be covered [24, 30, 31].

The relationships and connections journalists form on Twitter may influence the construction of their journalistic values, and ultimately influence news content [9, 32]. Further, the news values embodied by news content have downstream effects on news consumers behaviors [33]. In short, social and professional networks influence the environment in which journalists work, on news production [34], and on the consumption of the news.

## Network connections and positions, and their impact on news production

Given the importance of social media networks to the journalism profession, our work examines the distribution of journalism values and political ideology across journalists’ networks. In addition, we also examine how the size of the paper where journalists work relates to their position in the overall network. Below we explain how each of these network traits has important theoretical implications for news production and we draw on both literature from journalism and literature from the study of social networks to lay out our expectations.

### Professional connections and journalistic values

Social norms are commonly used rules that govern individuals’ actions and behaviors often through social pressure or expectations. Unlike other professions, journalism lacks a formal code of ethics or professional guidelines where violations would result in formal discipline. Instead, many journalistic behaviors are governed by ethical norms or journalistic values within the profession [28] and have a strong influence on journalists’ behavior [35]. More specifically, the Society of Professional Journalists identifies four principles of ethical journalism and encourages journalists and institutions to use them in practice [36]. These four principles include seeking and reporting truth, minimizing harm, acting independently, and being accountable and transparent [36]. These four principles of ethical journalism lay the foundations for the ethical values we examine in this paper, namely, truthfulness, accuracy, limitation of harm, objectivity, public accountability, and avoidance of political or ideological bias. Among these ethical values, truthfulness, accuracy, and no political bias tap into the general norm of objectivity that has governed journalistic practice [37]. These internally held beliefs help shape what reporters view as responsible reporting and help explain the decision of journalists to include or exclude stories from their coverage [24, 30, 38].

However, those ethical norms often vary across journalistic circles or between journalists working at the same outlet [39, 40]. As argued by Schudson [37], for ethical norms such as objectivity, above and beyond social control exerted by organizations and institutions, horizontal solidarity within journalists plays a key role in promoting objectivity. Historically, with the expansion of the journalism industry, the need for a cohesive group identity that would distinguish journalists from other professions prompted journalists to identify and develop professional norms, and in particular, the objectivity norm [37]. Though there is no doubt that news organizations play a key role in shaping journalists’ ethical values, it is conceivable that journalists’ professional networks and connections with each other contribute to the assimilation of these ethical values through the two mechanisms we will explain in the following paragraphs.

We examine the distribution of journalistic values within journalists’ networks and whether journalists holding similar values are more likely to be connected. There are two mechanisms which suggest journalists with similar journalistic values are likely to form relationships on Twitter. First, research on journalists has argued that reporters discover and internalize the ethical norms of other reporters in their network [24, 30, 37]. Journalists read the work of other journalists to identify newsworthy stories and to learn how to report them [32]. This process may contribute to the internalization of journalistic values, laying foundations for intermedia agenda-setting—a phenomenon where the norms of journalism lead to the homogenization of news coverage [25].

Second, similar journalistic values may lead journalists to build professional relationships on Twitter. Literature on social networks shows that homophily—the tendency for individuals to form ties with those sharing similar characteristics—often explains tie formation [26]. Previous work studying journalists’ networks shows that journalists tend to form connections based on gender, organization, and geographic location [23]. Though previous work focuses on homophily based on demographics, it is possible that similarity in journalistic values may drive relationship formation. All of this leads to our first hypothesis.

*H1*: Journalists are more likely than chance to be connected to other journalists holding similar journalistic values.

### Value homophily based on political ideology

Just as journalistic values influence news production, we might also expect ideological clustering (or lack thereof) within journalists’ networks to affect news exposure and judgements. Journalists’ interactions with other journalists who may have different viewpoints can have significant effects on news content [41]. Without exposure to different viewpoints journalists are more likely to write news stories in ways that reflect their own biases and ideological perspectives [42]. Homogeneous political environments can breed political extremism, foster opinion polarization, and lead to group-think [43, 44]. In contrast, the ideological diversity within a network increases knowledge of other political views and promotes greater political tolerance [45]. Understanding the distribution of ideological perspectives within the social networks of journalists enhances our understanding of the threat of ideological echo chambers.

We do not have clear expectations about the extent to which journalists form connections with other journalists based on similarity in political ideology. While individuals with similar traits tend to form ties at a higher rate than those with dissimilar traits [26], the extent to which individuals form connections based on political ideology remains empirically debated and is context-specific. While there is some evidence of ideological echo chambers on social media, this work has focused on ideological clustering among the public rather than in professional networks such as those of journalists [46–48]. Moreover, work has not found the sort of systematic ideological bias in what journalists choose to cover or how they cover news that would suggest a lack of exposure to other ideological viewpoints [49, 50]. Although the lack of ideological bias in news coverage might not necessarily indicate a lack of ideological clustering in journalists’ professional networks, it is conceivable that journalists might maintain connections with journalists with divergent viewpoints in order to expose themselves to different views in the news gathering process. As a result, we raise a research question about whether networks of journalists are clustered by ideology.

*RQ1*: Are journalists connected to other journalists with similar political ideologies more frequently than expected by chance?

### Centrality in networks and newspaper affiliation

Perhaps most importantly, we examine the network of journalists to document the centrality of journalists working at small and large papers in that network. Writing more than a half century ago, Breed [15] documented the tendency of editors to look to larger papers “to see how they handle the news.” As journalists engage in this process of intermedia agenda setting [25], they validate the newsworthiness of news stories and imitate how journalists at larger papers cover stories. While current journalists do not appear to have the same preference for news published in large papers over previously unpublished stories as Crouse [16] documented, previous work relying on survey experiments suggests that journalists continue to discount news published in smaller outlets relative to larger outlets [51] and news outlets continue to monitor the coverage of other outlets [30, 52] (Crouse [16] notes that some journalists would give partial stories to other larger papers to convince their editors to publish their full story). Together, previous work on journalism suggests that the size of a journalist’s employer may explain how much attention their work receives from journalists elsewhere.

Understanding the network in which reporters operate and how that network is related to reporters’ affiliations at large and small papers is important because it helps illuminate the mechanisms by which news flows through the media system [15, 51]. Previous work has argued that the choices of media outlets about what to cover and how to cover it are dependent on the choices of larger news institutions [15, 16, 30, 53, 54]. Inherent in this argument is the idea that journalists look to others at larger outlets about what to cover and how to cover it. While there has been some examination journalistic aping, previous work is limited in scope, focusing on small numbers of media outlets—mostly elite media—and looking at dyadic relationships rather than reporters’ relative positions in the larger professional network.

Furthermore, while studies have shown homogenization and content similarity at the level of news media [25], no studies have examined the underlying premise of intermedia agenda-setting—whether journalists’ professional networks reflect the tendency to look to others at larger outlets. Our work does this by using a large sample of journalists and information about their relationships to other journalists—working at other media outlets both large and small—to understand what news perspectives journalists are exposed to. Overall, we expect that journalists working for larger newspapers tend to hold more central positions in their professional networks than those working for smaller newspapers. Therefore, we propose the following hypothesis:

*H2*: Journalists employed at larger newspapers are more likely to be more central to the network of journalists on Twitter than journalists who are employed at smaller newspapers.

## Materials and methods

### Data

To test our hypotheses, we combined journalists’ network data on Twitter with survey data in which a sample of journalists reported their political ideology and ethical values. More specifically, by combining both network data and survey responses, we examined the role of network connections on journalistic values among 247 journalists (the number who completed both the survey questions about journalistic values and for whom we had Twitter handles), translating into 604 dyads, as well as the role of these connections on political ideology among 264 journalists (the number who completed the survey questions about political ideology and for whom we had Twitter handles), translating into 528 dyads. Finally, using the network data, we examined the relationship between the network positions of journalists and their newspaper size for 4,661 journalists for whom we had information about the frequency of publication or circulation and for whom we also had Twitter handles. While we had put forth earnest efforts to collect comprehensive data, for various reasons that were beyond our control, such as journalists’ lack of response to or failure to complete key questions on our survey, journalists who were not on Twitter, and the restrictions of Twitter API, the final numbers of cases being analyzed were smaller than the number of journalists in our sampling frame. In the following sections, we will explain the process by which we collected the data and the various ways in which we examined the data, including comparisons of sample characteristics across different subsamples being used. In the discussion section, we will return to this topic and discuss these limitations.

#### Survey data

To examine the values and ideologies of journalists, we rely on survey data of journalists working in political journalism in 2017 (Prior to collecting data on journalists, the research received IRB approval at [redacted]. We obtained consent from survey participants in the written format by asking participants to provide their consent in an online survey questionnaire). We identified over 13,500 political journalists working in newspapers. Given the role of the media in the nationalization of politics [55], the focus on political journalists was intentional. All identified journalists were invited to participate in the survey by email in late August and early September 2017. The survey received 1,507 responses for a response rate of 13.1%, although response rates to individual questions, including questions about ideology and ethics, were lower. However, response rates are similar to surveys of political elites [56] and almost double other surveys of journalists [12]. Moreover, the characteristics of the sample are representative of the universe of U.S. journalists and similar to other representative samples of journalists [57]. More details about the sample of journalists, the survey, and the respondents are available in S1 File.

#### Twitter network data

We operationalized journalists’ professional network ties on Twitter by capturing which journalists they follow (i.e., followee or “friend”) and by whom they are followed (i.e., “follower”) on Twitter. Twitter data were collected in compliance with the terms and conditions of data access at the time of data collection. Due to API constraints, we collected friends for all journalists and followers for those who completed the survey. After filtering out non-journalists, we recovered 135,314 friending and 16,724 following relationships among all journalists. Finally, we merged network data with survey data to examine clustering of journalists by ideology and journalistic values, resulting in roughly 500 to 600 dyads with valid responses. More details about Twitter data can be found in S1 File. We considered the potential biases introduced by missing data and found minimal evidence that these samples were systematically different, and these comparisons can be found in S1 File.

## Measures

The survey questionnaire included questions about journalists’ ideology and their journalistic values. Below we provide details on measurements and descriptive statistics for journalists who were surveyed and had Twitter accounts. More details and descriptive statistics for all variables of interest can be found in S1 File.

### Journalistic values

In the survey, we asked journalists the importance to them of six different journalistic values: “truthfulness,” “accuracy,” “limitation of harm,” “objectivity,” “public accountability,” and “avoiding political or ideological bias.” While there are likely a number of possible professional values that journalists might prioritize or consider in their reporting, we chose these because they are common factors that journalists consider in their work [37, 50, 58]. Similarly, because of widespread discussion about the importance of these values or norms in journalism, they are concepts journalists would find relatively easy to understand and can be conveyed using simple survey questions.

The importance of each of these items to journalists was assessed on the survey using a eleven-point scale with the end points labeled “extremely unimportant” (0) and “extremely important” (10). Among all six journalistic values, almost all journalists regarded truthfulness, accuracy, and public accountability to be highly important, resulting in little variance (Truthfulness: M = 9.92, SD = 0.34; Accuracy: M = 9.84, SD = 0.82; public accountability: M = 9.43, SD = 1.03). There was, however, substantial variation in evaluations of the other three items: limitation of harm (M = 8.02, SD = 2.13), objectivity (M = 8.85, SD = 1.83), and avoidance of political or ideological bias (M = 8.63, SD = 2.04). As such, our analyses below focus on the latter three journalistic norms because regression models cannot precisely estimate relationships outcome variables have little variation.

### Political ideology

To measure political ideology, we asked journalists to describe their personal political ideology on a five-point scale, ranging from “Very liberal” (1) to “Very conservative” (5). Among journalists who completed the survey and for whom we collected Twitter data, consistent with previous large-scale surveys of journalists [57], journalists surveyed tend to be moderate, leaning liberal (M = 2.43, SD = 0.82). A plurality of journalists (49.6%) identified themselves as strong liberals or liberals and a relatively smaller percentage (44.4%) self-identified as moderate, whereas a smaller percentage (6.0%) identified as very conservative or conservative.

### Journalists’ role and newspaper size

We coded every journalist’s role in the newspaper (i.e., reporter or editor). After accounting for dual journalistic roles and working for multiple newspapers, 58.0% were reporters whereas 42.0% were editors among all journalists with Twitter accounts (A few journalists acted as both an editor and a reporter. In the situation of dual roles, we retained the more senior role (i.e., editor). A few journalists in our sample were listed as staff for more than one newspaper simultaneously, which might result in more than one newspaper size for that journalist if data are available. In these few instances, we kept the larger newspaper size as the size of the newspaper(s) at which the journalist worked).

We used two different proxies for newspaper size—circulation size and frequency of publication of the newspaper—based on data from MondoTimes.com. Circulation size is an estimate of the average number of copies per publication. As circulation size was positively skewed, we took the natural logarithm of the variable (*M* = 9.63, *SD* = 1.58).

Our second measure is frequency of publication. This measures the frequency at which the newspaper publishes, ranging from “less than once a month (usually quarterly)” (1), “less than once a week (usually once a month)” (2), “once a week” (3), “more than once a week, but less than 5 times a week” (4), to “daily” (5). Among journalists with Twitter accounts, most journalists worked at daily newspapers (74.53%) and weekly newspapers (20.62%), whereas significantly fewer journalists worked at newspapers that published more than once a week (4.10%), monthly newspapers (0.64%), or quarterly newspapers (0.11%).

Although conceptually circulation size is more indicative of newspaper size, we were only able to identify the circulation size of the newspapers for a small subset of journalists (442 journalists or 7.75%). In contrast, frequency of publication data is available for the newspapers of 4,661 journalists in our sample (81.73%). As might be expected, these measures are moderately correlated (*r* = 0.49, *p* < 0.05). However, to be confident that the results are not an artifact of the way we measure newspaper size or biased because of missing data, we use both circulation size and frequency of publication in our analyses.

### Betweenness centrality

To quantify the centrality of journalists in diffusing information, we used betweenness centrality. Betweenness centrality measures the extent to which a node acts as a “broker” between different segments of the network so that information transmission would have been cut off if had the node been removed from the network [27]. A higher betweenness centrality score indicates a node has a stronger brokerage position in the network, playing a more significant role in transmitting information through the entire network.

We used the directed network to create a measure of betweenness centrality. Due to zeros, we added a constant to all betweenness centrality scores so that the minimum of the new variable equaled 1. This is an approach commonly used to deal with zero values on measures like betweenness centrality. As the variable was heavily skewed due to a number of journalists with high betweenness centrality scores, we took the natural logarithm of the variable.

## Analysis plan

For H1 and RQ1, we used generalized estimating equations (GEE) to account for multiple observations of the same ego across ego-alter pairings [59]. As journalistic values were correlated and the network ties were binary (i.e., whether a journalist follows another journalist or not), we deemed GEE as more appropriate than other methods such as MRQAP [60]. For H1, We estimated two GEE models separately on the journalist-friend pairings and the journalist-following pairings as we expect that friends’ journalistic values would predict an ego journalist’s values, whereas followers’ journalistic values would not predict an ego journalist’s values.

To test H2, we ran regression models, in which log-transformed betweenness centrality was predicted by the journalist’s role in the newspaper and a measure of newspaper size (either circulation or frequency of publication).

## Results

### Homophily by journalistic values

To test H1, we used GEE regression models to regress focal journalists’ values on their friends’(and followers’) values, controlling for whether the journalists in a dyad worked at the same newspaper.

As shown in Table 1, journalists tended to *friend* other journalists whose journalistic values were more similar to themselves than expected by chance. The more one’s friends perceived a journalistic values to be important, the more the focal journalist viewed it as important. The positive relationships held for limitation of harm, *b* = 0.11, *χ*^2^(601) = 8.10, *p* = 0.004, objectiveness, *b* = 0.07, *χ*^2^(601) = 4.06, *p* = 0.044, and avoidance of political or ideological bias, *b* = 0.11, *χ*^2^(601) = 5.35, *p* = 0.021, and the relationships were significant, after accounting for whether two journalists in a dyad worked at the same paper.

**Table 1:** The relationship between journalists’ journalistic values and their Twitter friends’ journalistic values.

	Limitation of harm	Objectiveness	No political/ideological bias
(Intercept)	6.987(0.428)***	8.116(0.392)***	7.536(0.536)***
Limitation of harm (friends)	0.109(0.038)**		
Objectiveness (friends)		0.068(0.034)*	
No political bias (friends)			0.111(0.048)*
Same paper	−0.030(0.330)	0.224(0.262)	0.047(0.296)
Scale parameter: gamma	4.792	3.170	3.991
Scale parameter: SE	0.918	0.753	0.837
Number of observations	604	604	604
Number of clusters	246	246	246

Unstandardized coefficients are reported and standard errors are in parentheses.

***p < 0.001;

**p < 0.01;

*p < 0.05.

Similarly, we found that journalists and their *followers* shared similar views on the importance of journalistic values. As shown in Table 2, the more important a particular journalistic value was to a journalist’s followers, the more important it was to the focal journalist as well. The positive relationships between focal journalists and followers were significant for limitation of harm, *b* = 0.11, *χ*^2^(543) = 4.79, *p* = 0.03, and avoiding political bias, *b* = 0.12, *χ*^2^(543) = 6.30, *p* = 0.01, but did not reach conventional levels of statistical significance for objectiveness, *b* = 0.07, *χ*^2^(543) = 3.02, *p* = 0.08.

**Table 2:** The relationship between journalists’ journalistic values and their Twitter followers’ journalistic values.

	Limitation of harm	Objectiveness	No political/ideological bias
(Intercept)	7.173(0.493)***	8.231(0.381)***	7.700(0.454)***
Limitation of harm (followers)	0.107(0.049)*		
Objectiveness (followers)		0.070(0.040)	
No political bias (followers)			0.118(0.047)*
Same paper	−0.073(0.290)	0.171(0.202)	−0.110(0.219)
Scale parameter: gamma	4.309	3.005	3.250
Scale parameter: SE	0.472	0.810	0.470
Number of observations	546	546	546
Number of clusters	226	226	226

Unstandardized coefficients are reported and standard errors are in parentheses.

***p < 0.001;

**p < 0.01;

*p < 0.05.

Our results did not provide evidence regarding the directionality of Twitter relationships, but this is because most journalists’ relationships in the data were mutual friendships. However, our results show that journalists tended to be more connected through social media to other journalists who shared the same journalistic values. Journalists were more likely to follow other journalists who had similar sets of journalistic values, even after controlling for shared employment. These findings support the expectation that journalists’ values come from networks that shape the professional expectations that journalists have (We also considered the possibility that the presence of similar journalistic values could be caused by similarities in product produced, regional norms, or the type of paper that an individual worked at (or in other words, that the relationship we find could be a proxy for the fact that journalists follow reporters with similar jobs where norms are a product of the environment where they worked). If we add these controls, none of them are significant with the exception of state dummy variables. The results for the relationship between journalists’ values and the values of their friends and followers remains the same. However, in a few instances (mostly the relationships between their values and the values of their followers) they no longer quite reach standard levels of statistical significance, although in many cases they are close. This is likely because many of these control variables are both conceptually similar (being measures of the environment in which journalists work) and empirically correlated to follower measures. These results with additional controls are reported and discussed in S1 File).

### No evidence of homophily by political ideology

In addition to journalistic values, we are also interested in the ideological clustering of journalists. To examine whether journalists tended to be friends with or followed by those with similar political ideologies, we examined the relationship between one’s ideology and the average ideology of one’s friends and of one’s followers. Should there be homophily based on political ideology, we would expect the average ideology of liberal journalists’ friends and followers to be liberal and the average ideology of conservative journalists’ friends and followers to be conservative.

We found no evidence of ideological homophily; instead, journalists tended to make friends with other journalists on Twitter irrespective of their political ideology. From extreme liberals to moderates, the average ideology of their friends tended to range from very liberal to moderate, with a few journalists making friends with conservatives. It is important to note that there were relatively few journalists who identified as conservative overall, however, their friends tended to range from liberal to moderate on average as well. The pattern held true for both the friending pattern and the following pattern (see S1 File).

Using GEE, we regressed the focal journalist’s political ideology on the ideology of the journalist’s friend’s or follower’s political ideology. As Table 3 shows, journalists’ political ideology was predicted neither by friends’ political ideology, *b* = 0.003, *χ*^2^(525) = 0.01, *p* = 0.93, nor followers’ political ideology, *b* = -0.002, *χ*^2^(479) = 0.00, *p* = 0.97. Again, the results of the journalist-friend pairings and the journalist-follower pairings overlapped given that most journalists whose political ideology scores were observed were mutual friends.

**Table 3:** The relationships between journalists’ ideology and their Twitter friends’ and followers’ ideology.

	Journalists’ Ideology	
(Intercept)	2.465(0.117)***	2.447(0.127)***
Political Ideology (Friends)	0.003(0.037)	
Political Ideology (Followers)		−0.002(0.047)
Working for same paper	−0.147(0.087)	−0.096(0.092)
Scale parameter: gamma	0.520	0.597
Scale parameter: SE	0.046	0.055
Number of Observations	528	482
Number of clusters	231	215

Unstandardized coefficients are reported and standard errors are in parentheses.

***p < 0.001;

**p < 0.01;

*p < 0.05.

Overall, our results suggest that, unlike journalistic values, journalistic networks were not clustered on the basis of ideology. Journalists were not less (or more) likely to make connections on social media with other journalists who had different (the same) ideological predispositions. Within the profession, our results suggest that journalists are not isolating themselves from other journalists on the basis of ideology.

### Journalists from larger newspapers are more central in the network

**Fig 1:**
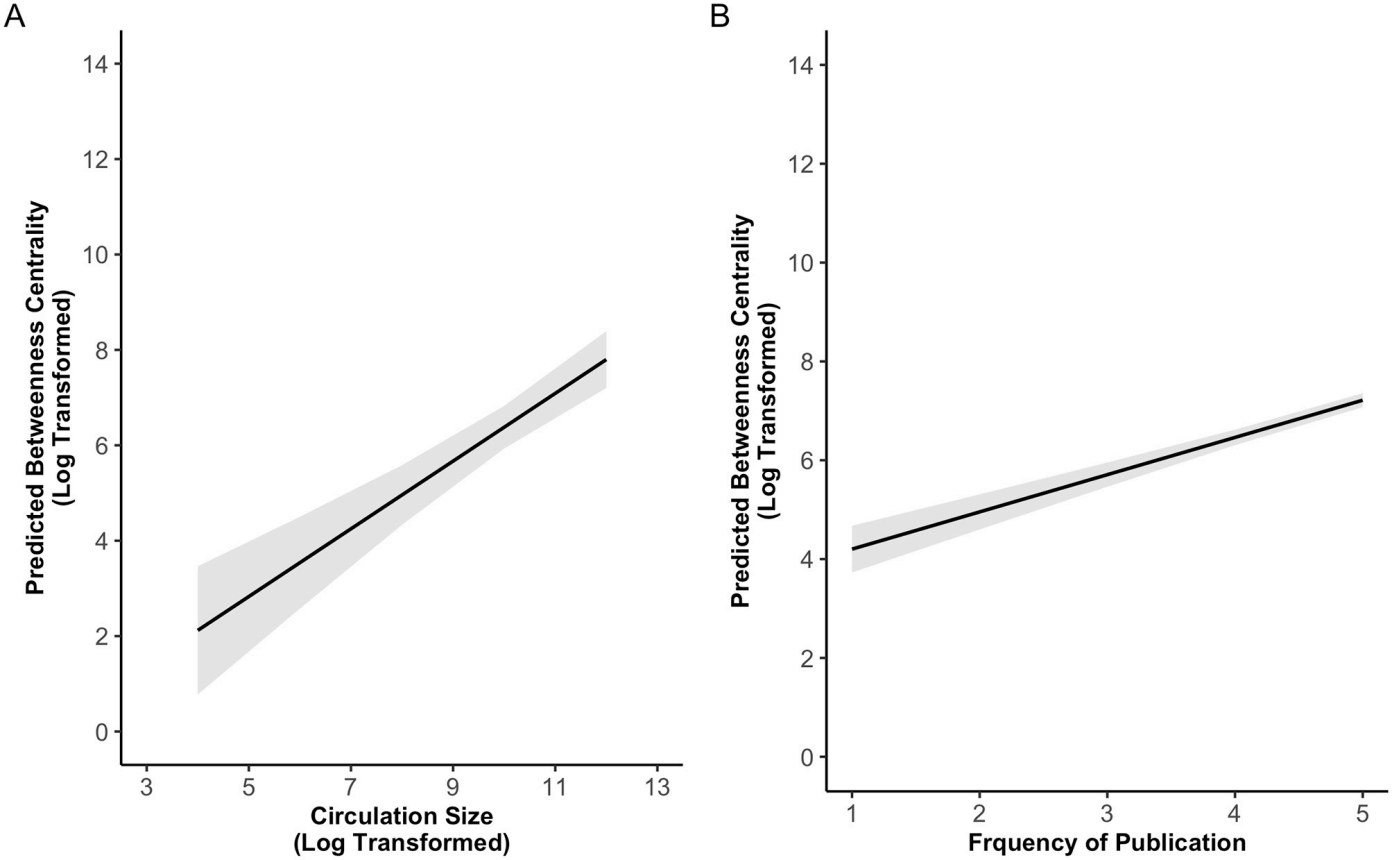
The relationship between betweenness centrality and newspaper size. Predicted log-transformed betweenness centrality at varying levels of log-transformed circulation size (panel A) and frequency of publication (panel B).

Lastly, we are interested in the amount to which journalists at larger newspapers are more central to the professional network. Table 4 and Fig 1 shows the results of regression models predicting log-transformed betweenness centrality by both the position of a journalist and the two indicators of newspaper size. As shown in the model on the left of Table 4 and in Panel A of Fig 1 using circulation size as the proxy, overall, the model explained 9.8% of the variation in log transformed betweenness centrality. After accounting for the position the journalists’ held (*b* = -0.196, *t*(439) = -0.56, *p* = 0.58) the circulation size of the newspaper for which a journalist worked was positively associated with the journalist’s betweenness centrality on Twitter (*b* = 0.710, *t*(439) = 6.71, *p* < 0.001). To put the coefficient into context, a 1% increase in circulation size was associated with approximately 0.71% increase in a journalist’s betweenness centrality on Twitter.

**Table 4:** The relationship between betweenness centrality and newspaper size.

	Log-transformed Betweenness Centrality	
(Intercept)	−0.720(1.094)	3.448(0.302)***
Editor	−0.196(0.353)	−0.505(0.108)***
Log of circulation Size	0.710(0.106)***	
Frequency of publication		0.753(0.063)***
R^2^	0.098	0.039
Adj. R^2^	0.094	0.039
Number of observations	442	4, 661

Results of regression models predicting log-transformed betweenness centrality by one’s role in a newspaper (editor or reporter) and log-transformed circulation size (left) or frequency of publication (right). Unstandardized coefficients on the logarithmic scale are reported.

***p < 0.001;

**p < 0.01;

*p < 0.05.

Likewise, the model on the right of Table 4 and Panel B of Fig 1 shows that, when we used frequency of publication as the proxy of newspaper size, there was a significant positive relationship (*b* = 0.753, *t*(4,658) = 11.92, *p* < 0.001). The positive relationship between journalists’ betweenness centrality in the Twitter network and their newspaper size was robust to alternative data transformation (see S1 File). The nearly identical results, as shown in both Table 4 and Fig 1 using two different proxies bouy our confidence that our results are not skewed by which newspapers make circulation data available.

## Discussion

### General discussion

The choices that journalists make about what to cover and how to cover it have a meaningful influence on society. To understand these dynamics, we must understand the environment in which journalists operate professionally. This environment is increasingly influenced by social media, particularly the Twitter networks that journalists inhabit. Our work examines this online professional environment and the network in which journalists are situated to better understand the relationships between journalists in their professional environment.

First, our results show that the professional network in which journalists are embedded is highly correlated with the journalistic values they hold. We find that journalists are more likely to be connected to other journalists who have similar views of the importance of objectivity, limitation of harm, and the avoidance of political or ideological bias. This finding is consistent with previous work showing reporters discover and internalize the journalistic values of what is appropriate to cover and how to cover news [24, 30, 37], as well as the homophily-based explanation [26]. Moreover, such correlations of journalistic values between people connected on Twitter exist after accounting for the role of news outlets, suggesting that journalists’ online professional network is unique in its own right [32].

Notably, however, we do not find a similar clustering of journalists by political ideology. While conservative journalists are underrepresented in journalism, we do not find that conservative journalists are isolated. Likewise, while journalism is dominated by journalists with liberal political views [10, 50], liberal reporters are not further isolated from conservative viewpoints in the network. The presence of ideological integration provides some assurance that as they build their professional networks, journalists, at least those who work for newspapers, are not intentionally isolating themselves from diverse viewpoints in a way that might extenuate political extremism and polarization. This is consistent with the evidence showing the lack of an ideological bias in news coverage [49, 50]. In an effort to limit the ideological bias in what and how news stories are covered, journalists—regardless of their political ideology—maintain connections with those who have divergent viewpoints.

Last but not least, we find that journalists shape the professional environment in which they work in a way that directs journalists to look to larger papers. Our work provides evidence for the premise of intermedia agenda-setting [25]. Consistent with work that found journalists tend to discount news published in smaller media outlets relative to larger media outlets [15, 51], we find that journalists at larger papers have greater brokerage power over the entire network than journalists at smaller papers. Our finding comports with the concept of journalistic capital or editorial capital, which is the specific, cultural capital cultivated by the journalists in the field, encompassing their professional experience, their organizational positions, news beat, among others [61–63]. More specifically, our finding suggests that journalists who work for larger newspapers have accumulated more journalistic capital over time owing to their professional experience in larger, more prestigious newspapers compared with those who work for small newspapers. The journalistic capital cultivated by journalists are manifested by their network positions, and in particular, the centrality of these journalists within the profession. We note that there are other reasons for why journalists from larger papers are more central to journalists’ professional networks than those from smaller papers. We will return to this topic and elaborate on opportunities for future research in a later section.

This finding has implications for what news stories journalists might encounter in the social media environment. This pattern of attentiveness to journalists working in larger newspapers magnifies the effect of discounting news from small outlets. Not only are journalists more likely to discount news from smaller outlets, they are also less likely to notice it as a result of their online professional environment. Journalists are more likely to see information shared by journalists at larger outlets than their own and less likely to see news shared by journalists working at smaller outlets. Our findings explain the methods by which news tends to spread from larger outlets to smaller ones [15, 30, 52, 53].

Our work focuses on the meso-level analysis of news production by examining journalists’ professional networks. Though our study does not examine how journalists’ networks impact the news stories they produce [21], we shed light on journalistic networks to better understand the journalistic values they hold, the stories they see, and ultimately the news they produce.

We provide several important insights into the professional environment in which journalists work. First, we show that journalists are connected with other journalists based on shared journalistic values on Twitter. The professional environment on social media in which journalists operate is highly correlated with their own professional values, even after accounting for the outlets where they work. Second, we examine if journalists build their professional networks on Twitter based on political ideology. However, we find no evidence that journalists are connected with each other on the basis of their political ideology. The lack of evidence for ideological clustering among journalists on social media suggests journalists pay attention to other journalists regardless of their political ideology. Finally, we add holistic, quantitative evidence to the phenomenon of “aping” of larger outlets [15, 16], which is the premise of intermedia agenda-setting [25], as well as the concept of journalistic capital or editorial capital [61–63]. We show that journalists employed at smaller newspapers tend to “peer over the shoulder” of those employed at larger newspapers in that journalists at larger newspapers tend to be more central and have greater brokerage power. Our finding provides real world observational evidence corroborating findings that journalists place greater value on news reported at larger outlets [15, 16, 51].

### Limitations and future research

Our work is not without limitations. First, we would caution readers that our evidence is largely correlational. We attempt to evaluate the directionality patterns in journalists’ values and ideology. However, given the large proportion of mutual connections and the small number of directional relationships, we are unable to ascertain whether the observed relationship in journalists’ values stems from socialization, homophily, or both—a question that merits more empirical research.

Second, our study suffers at times from missing data in a key variable, i.e., self-reported political ideology. Although not related to concerns about expressing ideology, some journalists exited the survey prior to viewing the question on political ideology. (The number of respondents who declined to provide their ideology after having answered the question in the survey immediately prior to the question about their ideology—which was about whether they were full-time, part-time, or volunteer in their position—was only 31 respondents (3%) out of 927.) The early exit of some respondents to the survey means that our analysis of ideological clustering is based on a smaller sample of journalists who completed the survey, which could result in insufficient statistical power. However, as the distribution of political ideology among journalists in our sample is consistent with that of the larger survey of journalists, and as those who provided political ideology were not systematically different from the entire sample (see “Potential Biases in the Survey and the Twitter Sample” in the S1 File), our conclusion regarding ideological clustering, despite the small sample size of conservatives and extreme conservatives, provides important evidence that future researchers may build upon. Future research should consider ways to overcome these obstacles either through other valid measures of political ideology or through other behavioral measures [21].

Third, due to constraints in accessing Twitter data, our data consist of the friend lists of all political journalists on Twitter, including both survey-takers and non-survey takers, but yet the follower lists only include those who participated in survey. This is because journalists tend to have more followers than friends and some journalists may have thousands of followers, which slows down and even breaks our data collection process. As a compromise, we focused on collecting friend lists of all political journalists. The decision does not influence the directed network of journalists used for testing RQ1 and H2 much, though it may have some influence on H1 because the following dyads may be incomplete.

Importantly, while we follow best practices in creating our own list which allows for greater quality control and perform a census of the sampling frame [64], our work focuses specifically on political newspaper journalists. In particular, our study only covers journalists who were directly employed by newspapers, reported politics, and maintained a public presence on Twitter. While the survey population is comparable to other representative surveys of journalists, it could be that journalists outside of the scope of the sample we use could be unique and might be different. Indeed, newspapers are legacy organizations with particularly strong institutional norms and values and journalists working in those venues could have a heightened awareness of those values relative to other journalists. Nor do we study how newspaper journalists build professional connections with journalists working for other media genres, such as television. Thus, it is unclear how our conclusions can be extrapolated to all journalists—a question that merits more scrutiny in future research. Indeed, in particular, we note that recent work has noted key differences in how journalists at mainstream publications and journalists at alternative media behave. While there is little variation in some values, network effects could be even stronger if we were to widen the cross-section of journalists to include journalists at other media outlets beyond newspapers.

Despite these limitations, our work provides clear insights into the professional networks of newspaper journalists who report politics and establishes pathways for future research in this area. Our finding suggests that journalists who work for larger newspapers tend to possess more central positions in journalists’ networks than journalists working for smaller papers. This finding is consistent with the prior work on “aping”, “peering over the shoulder” [15, 16], and the concept of journalistic capital [61], but there might be other reasons for why journalists from large papers tend to be central. For instance, it is possible that larger newspapers tend to have more resources, which translate into the higher quality of news coverage and the greater attention from journalists from other newspapers. It is also conceivable that larger newspapers are better able to set the agenda, leading government officials, audiences, and other media outlets to follow the agenda. Though we are unable to test these mechanisms using our data, our finding of the centrality of journalists at larger papers lays the groundwork for future research to separate out these processes more thoroughly.

## Conclusion

Understanding the professional networks of journalists on social media provides important insights into the environment in which journalists work. Overall, journalists working at larger media outlets are more central to the professional networks of journalists. We show consistent patterns of journalistic “aping” or “peering over the shoulder” where journalists follow (are followed by) other journalists at larger (smaller) news outlets in a way that confirms previous arguments about where journalists look to find news and the value that they place on news from smaller news outlets. This finding also squares with the idea of “journalistic capital” [61], in which journalists’ positions in networks reflect their social capital, creating unique advantages to those who hold more central positions. Our evidence also indicates that above and beyond many other mechanisms that shape journalists’ values, journalists’ connections with other journalists in the profession may contribute to the professional norms and values they hold. We do not, however, find the same clustering of ideological preferences, suggesting that while journalists are adopting the journalistic norms of the professional community in which they work, they are not selecting that community on the basis of ideological preferences. On the whole, our work provides a better understanding about the nature of journalistic networks and its relationship to the creation and dissemination of political news.

## Supporting information

S1 File Supplementary appendix. (PDF)
