# Supplementary material for: Journalists’ networks: Homophily and peering over the shoulder of other journalists
Source: PLoS One. 2023 Oct 18;18(10):e0291544. doi: 10.1371/journal.pone.0291544 (PMC10584149; doi:10.1371/journal.pone.0291544)
Supplement: S1 File — (PDF) [file pone.0291544.s001.pdf]

# Journalists' networks: Homophily and peering over the shoulder of other journalists

***(Supplementary materials for online publication)***

## S1 Appendix. Details on survey.

The sample for the survey was collected using U.S. Newspaper List (usnpl.com). U.S. Newspaper List is a comprehensive online media directory of over 4,000 newspapers (and a significant number of television and radio stations) operating in the United States. The website provides the address of each media outlet as well as links to each media outlets' website, Facebook, Twitter, and YouTube page. A team of researchers used this website between May of 2017 and July of 2017 and visited the website or Facebook page of every newspaper listed in each state to gather the email addresses of political editors, political reporters, and publishers. We identified political reporters and editors as those whose titles or job descriptions on the newspaper website included the word "politics" or "government" or a government body (e.g., "state house" or "city hall"). Publishers were included in the sample because initial conversations the research team had with small town media personnel suggested that for many small papers the publisher also had extensive input on editorial and content decisions. In cases where a specific reporter (or editor) or group of reporters (or editors) was not explicitly designated as being assigned to political news, the email addresses of all reporters and editors were collected. In most instances where contact information for all reporters was collected it was because the newspaper staff was small and reporters were not specialized. Discussions with staff at similar papers suggested most reporters covered the full gamut of news the newspaper covered. The process explicitly excluded reporters and editors designated as covering non-political news (e.g., lifestyle or sports). In addition, the data only includes individuals listed as newspaper staff and excludes freelance and nationally syndicated journalists whose reporting might appear in the newspaper.

Our efforts follow identified best practices in creating our own list which allows for greater quality control and perform a census of the sampling frame (as well as the practices of sending multiple reminders to participate (journalists were sent an original invitation to participate and two additional follow up emails), sending emails from trusted university domain email, using a

brief subject line ("[University] Survey of Journalists"), and providing a skimmable survey invitation which provided "a brief introduction of the sender; a very brief description of the project and its contribution to the practice of journalism; an invitation to participate in the survey with clear indications about the amount of time the survey should take and any incentives being offered; separate links to begin the survey and opt-out from it; a description of the confidentiality of responses; and a signature with contact information for the sender and lead researcher"Molyneux and Zamith, 2022.

We recognize, however, that sampling only newspaper journalists misses many individuals who would logically be classified as journalists, however, other aspects of this research project (published elsewhere) required the collection of news content which was more readily available for newspapers than for other news mediums. Similarly, other aspects of the research project were also focused specifically on political news which warranted our limiting our sample to journalists covering politics.

This process resulted in a population of over 13,500 newspaper journalists who were explicitly focused on covering politics and government affairs. Invitations to participate in the survey were sent by email in late August and early September of 2017. A total of 1,507 journalists responded to the survey for a response rate of 13.1%, although as noted previously, response rates to individual questions on the survey varied.

The survey asked journalists, editors, and publishers about a wide range of items related to politics and their responsibilities as journalists. In addition to the questions reported here, the other questions on the survey addressed how a journalist would cover particular stories and how newsworthy different types of political stories would be to editors and readers. Table A displays the descriptive characteristics of the sample. The vast majority of respondents are full-time journalists, with only 3% indicating they are volunteers or part-time employees. The survey received responses from respondents working at a variety of newspaper outlets, mostly midsized dailies and small local papers.

Table A shows the characteristics of the sample. Reassuringly, our sample is comparable to

other representative surveys of journalists. While we find that only 1.3% of respondents work at a (self-reported) national paper, this is representative of of the overall population of journalists. Ali and Radcliffe (2017) report 97% of U.S. newspapers have circulations of under 50,000. Similarly, in their 2013 study, Weaver et al. (2019) found the average age of journalists to be 41 which is very close to the average of 46 in our sample (more closely in line with these numbers, Cooper and Johnson (2009) find the average age of state house reporters is 45). Similarly, Weaver et al. (2019) found 37% of journalists are women compared to the 40% in our sample. In addition, with regards to journalist ideology, Weaver et al. (2009) find 40% of journalist describe themselves as "a little to the left" or "pretty far to the left" of the ideological spectrum which is almost identical to the 42% in our survey who identify as liberal or very liberal.

**S2 Appendix. Details on Twitter networks.**

To identify newspaper journalists on Twitter, the research team searched for every journalist (regardless of survey participation) in the sampling frame on Twitter using their name, the email address, or the newspaper in which they worked to identify their Twitter handles. 59.1% of journalists in the sampling frame were identified on Twitter and their handles were recorded. We collected their user profiles as well as lists of friends and followers on Twitter if accessible through the Twitter API. We tried to recover the network data as much as possible, but due to the constraints of API in limiting the amount of data accessible, we focused more on collecting data of friends than on data of followers given that journalists tend to have many more followers than friends. We excluded some inaccessible accounts, either because journalists made their account private—only allowing an approved follower to see their tweets—or had deleted their account by the time we collected their data in 2020. In total, we were able to collect the friend edgelists of 5,078 journalists and the follower edgelists of 720 journalists who responded to the survey and were on Twitter.

To construct the professional networks of journalists on Twitter, we searched through the friend and the follower edgelists to identify all political journalists in those edgelists who were in our sampling frame. As a result, the friending network consisted of 5,690 journalists, making up

83.3% of all political journalists on Twitter identified in our sampling frame. The following network constituted 4,150 journalists, making up 60.7% of alljournalists on Twitter. These journalists formed 135,314 friending and 16,724 following relationships with other journalists in the sample. Using the above data, we were able to create a measure of betweenness centrality to test H2.

Finally, we merged the network data with the survey data to examine the clustering of journalist ideology and journalistic values as hypothetized in H1 and RQ1. Our analysis focused primarily on editors and reporters, and therefore, we left out publishers from the pool of journalists. Our analysis of ideological clustering were based on 556 dyads of journalists and friends (who were also journalists) as well as 503 dyads of journalists and followers (who were also journalists). For analysis of journalistic value clustering, we analyzed 604 dyads of journalists and friends as well as 546 dyads of journalists and followers respectively.

**S3 Appendix. Potential biases in subsamples.**

Table B compares four groups of journalists—all newspaper journalists (n = 13,019), journalists whose Twitter accounts were identified (n = 7,667), survey takers (n = 1,507), and journalists who completed the entire survey (n = 895). Survey takers—both those who finished some questions and those who completed the entire survey—appeared to be very similar to all newspaper journalists in our sampling frame despite some slight differences. In terms of gender, we find only very small differences. On average, survey takers and those who completed the entire survey were only slightly more likely to be women—42.85% and 42.84% of the sample—relative to the full population of newspaper journalists (40.58%). Likewise, the demographic and political characteristics of the counties where journalists lived were very similar on average. Compared to the full sample, those who completed the survey lived in counties where on average there were insignificantly higher percentages of white people (78.15% for all survey takers and 78.89% for survey takers with full completion), and where rates of college degrees (30.45% and 30.03% respectively), unemployment (5.19% and 5.14% respectively), and Democratic vote share (44.49% and 43.49% respectively) were all nearly identical to the full sample.

Likewise, we report the comparison between all newspaper journalists and journalists on Twitter and find only small differences between samples. Women made up 40.17% of journalists on Twitter, compared with 40.58% for all newspaper journalists. The average percentage of white people in counties where journalist on Twitter lived was 75.39%, compared with 77.49% for all journalists. Journalists on Twitter appeared to live in counties where on average 32.31% had college degrees, slightly higher than that (30.36%) for all journalists. The unemployment rates in counties where journalists worked were very similar on average, with an unemployment rate of 5.32% for counties with journalists who were on Twitter and 5.28% for all journalists. Finally, journalists on Twitter appeared to live in slightly more Democratic counties on average (48.36%) than all newspaper journalists (44.99%). Overall, while there are some small differences, on the whole, these differences are not substantial and concerns of representativeness should not radically alter our conclusions.

**S4 Appendix. Descriptive statistics of key variables.**

Table C shows valid n, mean, standard deviation, minimum, and maximum of key variables used in our analyses.

**S5 Appendix. Raw data plots.**

Figure A shows political ideology of journalists and the average political ideology of their friends. Figure B shows political ideology of journalists and the average political ideology of their followers. To avoid overlapping of the data, we added a small degree of random noise to the variable of ideology of journalists on the x-axis.

**S6 Appendix. Sensitivity analyses for journalistic values.**

We report additional results for similarity in journalistic values. Due to the moderate correlation between some of the control variables and their conceptual overlap with each other, wed decided to report these results here for further reference.

Table D and Table E show the results of several models controlling for whether the pair of journalists worked for the same paper, had the same position in the newspaper, or worked for newspapers with the same frequency of publication. With these two additional control variables,

while the coefficients for friends' or followers' ethical values were positive and of the same magnitude as those from the original models, some coefficients reached the conventional statistical significance level whereas others not. In particular, the associations between journalists' and their friends' values on limitation of harm (b = 0.10, SE = 0.04, p = 0.01) and no political bias (b = 0.11, SE = 0.05, p = 0.04) were robust, but friends' values on objectiveness were not significantly related to journalists' values (b = 0.05, SE = 0.04, p = 0.23). Similarly, for journalists and their followers, while the relationship regarding objectiveness was not significant (b = 0.04, SE = 0.05, p = 0.41), the relationship regarding limitation of harm was significant and positive based on the one-tailed test of significance (b = 0.10, SE = 0.05, one-tailed p = 0.03) and the relationship regarding no political bias remained significant (b = 0.12, SE = 0.05, p = 0.02).

In Table F and Table G, we controlled for the above variables as well as whether journalists lived in the same state. As shown by the tables, the findings remain largely the same: while the coefficients remained positive, some of them failed to reach the conventional level of statistical significance. Specifically, when it comes to the similarity in journalists' and their Twitter friends' ethical values, while the relationships regarding objectiveness (b = 0.02, SE = 0.04, p = 0.71) and political bias (b = 0.08, SE = 0.05, p = 0.12) were insignificant though positive, the relationship in terms of limitation of harm was positive and significant based on the one-tailed test (b = 0.07, SE = 0.04, one-sided p = 0.04). For journalists' and their followers' ethical values, both the relationship regarding limitation of harm (b = 0.09, SE = 0.06, p = 0.11) and objectiveness (b = 0.02, SE = 0.06, p = 0.74) were insignificant, but the relationship regarding political bias was significant and positive (b = 0.10, SE = 0.05, p = 0.04).

However, we are hesitant to read too much into the findings here because, as we have detailed in the manuscript, two of the control variables—working for the same paper and living in the same state—overlapped with each other both conceptually and empirically.

**S7 Appendix. Sensitivity analyses for betweenness centrality.**

To examine whether the result with regard to log-transformed betweenness centrality is robust, we omitted journalists whose betweenness centrality equalled zero and estimated

regression models on the log-transformed betweenness centrality. As shown in both models in Table H, the coefficients for circulation size and frequency of publication remained significant and was in the same direction, though the size of the coefficients differed from that in the original models.

We also used rank positions of betweenness centrality and circulation size and calculated the Spearman's correlation coefficient between their rank positions. Rank positions and Spearman's correlation are used if variables are heavily skewed. The rank positions of these two variables have a monotonically increasing relationship, regarding of whether we averaged rank positions, $\rho = 0.325$, $p < 0.001$, or randomly assign rank positions, $\rho = 0.327$, $p < 0.001$, to handle duplicated ties.

**Table A.** *Sample Demographics.*

| Characteristics | Percentage of Sample |
|---|---|
| **Role at Paper** | |
| Reporter | 43.9% |
| Editor | 36.9% |
| Publisher | 11.1% |
| Other Role | 8.2% |
| **Paper Type (self classified)** | |
| National Paper | 1.3% |
| Midsized Paper | 27.3% |
| Small Town Paper | 65.6% |
| Specialty Paper | 5.8% |
| **Employment** | |
| Full-Time Journalist | 96.9% |
| Party-Time Journalist | 2.9% |
| Volunteer Journalists | 0.2% |
| **Ideology** | |
| Very Liberal | 12.1% |
| Liberal | 30.4% |
| Moderate | 45.7% |
| Conservative | 9.4% |
| Very Conservative | 2.0% |
| **Gender** | |
| Male | 56.8% |
| Female | 43.2% |
| Observations | 1,510 |

*Note:* The number of observations in the ideology, employment, and gender categories have smaller sample sizes (n=896, n=896, and n=927 respectively)

**Table B.** *Comparisons between different samples.*

| | All Journalists | Journalists on Twitter | Survey takers | Survey takers (who completed the entire survey) |
|---|---|---|---|---|
| n | 13,019 | 7,667 | 1,507 | 895 |
| Female | 40.58% | 40.17% | 42.85% | 42.84% |
| Average percent of white people (County) | 77.49% | 75.39% | 78.15% | 78.89% |
| Average percent of people with college degrees (County) | 30.36% | 32.31% | 30.45% | 30.03% |
| Average percent of unemployed people (County) | 5.28% | 5.32% | 5.19% | 5.14% |
| Average Democratic vote share (County) | 44.99% | 48.36% | 44.49% | 43.49% |

*Note*. Comparisons between all newspaper journalists, journalists on Twitter, and journalists who completed the entire survey. Variables except for gender are measured at the county level. Percentage of female journalists, and average percentages of white people, individuals with college degrees, unemployed individuals, or Democratic voters in counties where journalists lived are reported and compared across three samples.

**Table C.** *Descriptive statistics of key variables.*

| | n | Mean | SD | Min | Max |
|---|---|---|---|---|---|
| Political ideology | 401 | 2.43 | 0.82 | 1.00 | 5.00 |
| Truthfulness | 413 | 9.92 | 0.34 | 7.00 | 10.00 |
| Accuracy | 413 | 9.84 | 0.82 | 0.00 | 10.00 |
| Limitation of harm | 410 | 8.02 | 2.13 | 0.00 | 10.00 |
| Objectivity | 413 | 8.85 | 1.83 | 0.00 | 10.00 |
| Public accountability | 413 | 9.43 | 1.03 | 4.00 | 10.00 |
| Avoiding political or ideological bias | 413 | 8.63 | 2.04 | 0.00 | 10.00 |
| Betweenness centrality | 5703 | 12875.39 | 39000.74 | 0.00 | 1313871.00 |
| Log-transformed betweenness centrality | 4732 | 7.74 | 2.65 | -3.85 | 14.09 |

*Note*. All journalists with valid Twitter handles are included in the descriptive analysis. Betweenness centrality is calculated from the directed Twitter network.

**Table D.** *The relationship between journalists' journalistic values and their Twitter friends' journalistic values.*

| | Limitation of harm | Objectiveness | No political bias |
|---|---|---|---|
| (Intercept) | 7.452 (0.413)*** | 8.263 (0.393)*** | 7.594 (0.562)*** |
| Limitation of harm (friends) | 0.105 (0.041)* | | |
| Objectiveness (friends) | | 0.051 (0.042) | |
| No political bias (friends) | | | 0.109 (0.053)* |
| Same paper | 0.064 (0.387) | 0.176 (0.291) | 0.057 (0.353) |
| Same position | -0.292 (0.245) | -0.316 (0.204) | -0.118 (0.241) |
| Same frequency of publication | -0.319 (0.315) | 0.290 (0.401) | 0.031 (0.326) |
| Scale parameter: gamma | 4.815 | 2.923 | 4.030 |
| Scale parameter: SE | 0.936 | 0.751 | 0.878 |
| Num. obs. | 542 | 542 | 542 |
| Num. clust. | 218 | 218 | 218 |

***$p < 0.001$; **$p < 0.01$; *$p < 0.05$

**Table E.** *The relationship between journalists' journalistic values and their Twitter followers' journalistic values.*

| | Limitation of harm | Objectiveness | No political bias |
|---|---|---|---|
| (Intercept) | 7.453 (0.553)*** | 8.515 (0.454)*** | 7.692 (0.545)*** |
| Limitation of harm (followers) | 0.101 (0.054) | | |
| Objectiveness (followers) | | 0.043 (0.053) | |
| No political bias (followers) | | | 0.120 (0.052)* |
| Same paper | 0.012 (0.332) | 0.074 (0.170) | -0.162 (0.251) |
| Same position | -0.318 (0.197) | -0.221 (0.208) | -0.116 (0.220) |
| Same frequency of publication | -0.079 (0.290) | 0.225 (0.400) | 0.135 (0.256) |
| Scale parameter: gamma | 4.295 | 2.484 | 3.235 |
| Scale parameter: SE | 0.503 | 0.649 | 0.514 |
| Num. obs. | 487 | 487 | 487 |
| Num. clust. | 202 | 202 | 202 |

***$p < 0.001$; **$p < 0.01$; *$p < 0.05$

**Table F.** *The relationship between journalists' journalistic values and their Twitter friends' journalistic values.*

| | Limitation of harm | Objectiveness | No political bias |
|---|---|---|---|
| (Intercept) | 6.983 (0.506)*** | 8.041 (0.469)*** | 7.319 (0.620)*** |
| Limitation of harm (friends) | 0.074 (0.043) | | |
| Objectiveness (friends) | | 0.017 (0.045) | |
| No political bias (friends) | | | 0.078 (0.050) |
| Same state | 1.112 (0.554)* | 0.806 (0.485) | 0.837 (0.523) |
| Same paper | -0.423 (0.281) | -0.174 (0.204) | -0.314 (0.234) |
| Same position | -0.222 (0.227) | -0.269 (0.180) | -0.063 (0.222) |
| Same frequency of publication | -0.275 (0.305) | 0.333 (0.384) | 0.070 (0.302) |
| Scale parameter: gamma | 4.611 | 2.817 | 3.916 |
| Scale parameter: SE | 0.768 | 0.637 | 0.773 |
| Num. obs. | 542 | 542 | 542 |
| Num. clust. | 218 | 218 | 218 |

***$p < 0.001$; **$p < 0.01$; *$p < 0.05$

**Table G.** *The relationship between journalists' journalistic values and their Twitter followers' journalistic values.*

| | Limitation of harm | Objectiveness | No political bias |
|---|---|---|---|
| (Intercept) | 7.247 (0.553)$^{***}$ | 8.347 (0.480)$^{***}$ | 7.479 (0.565)$^{***}$ |
| Limitation of harm (followers) | 0.087 (0.055) | | |
| Objectiveness (followers) | | 0.019 (0.059) | |
| No political bias (followers) | | | 0.105 (0.050)$^{*}$ |
| Same state | 0.483 (0.241)$^{*}$ | 0.555 (0.345) | 0.497 (0.248)$^{*}$ |
| Same paper | -0.197 (0.336) | -0.163 (0.209) | -0.378 (0.240) |
| Same position | -0.285 (0.194) | -0.184 (0.194) | -0.078 (0.218) |
| Same frequency of publication | -0.054 (0.290) | 0.267 (0.401) | 0.167 (0.243) |
| Scale parameter: gamma | 4.258 | 2.436 | 3.196 |
| Scale parameter: SE | 0.500 | 0.596 | 0.500 |
| Num. obs. | 487 | 487 | 487 |
| Num. clust. | 202 | 202 | 202 |

$^{***}p < 0.001$; $^{**}p < 0.01$; $^{*}p < 0.05$

**Table H.** *The relationship between betweenness centrality and newspaper size.*

| | Log-transformed betweenness centrality | |
|---|---|---|
| | Model S1 | Model S2 |
| (Intercept) | 4.738 (0.815)$^{***}$ | 6.486 (0.249)$^{***}$ |
| Editor | -0.249 (0.261) | -0.358 (0.086)$^{***}$ |
| Circulation size (Log-transformed) | 0.313 (0.078)$^{***}$ | |
| Frequency of publication | | 0.318 (0.052)$^{***}$ |
| $R^2$ | 0.050 | 0.016 |
| Adj. $R^2$ | 0.045 | 0.016 |
| Num. obs. | 352 | 3960 |

*Note*. Results of regression models predicting log-transformed betweenness centrality by one's role in a newspaper (editor or reporter) and log-transformed circulation size (Model S1) or frequency of publication (Models S2). Journalists whose betweenness centrality scores equal zero are omitted from the models. Unstandardized coefficients on the logarithmic scale are reported. $^{***}p < 0.001$; $^{**}p < 0.01$; $^{*}p < 0.05$.

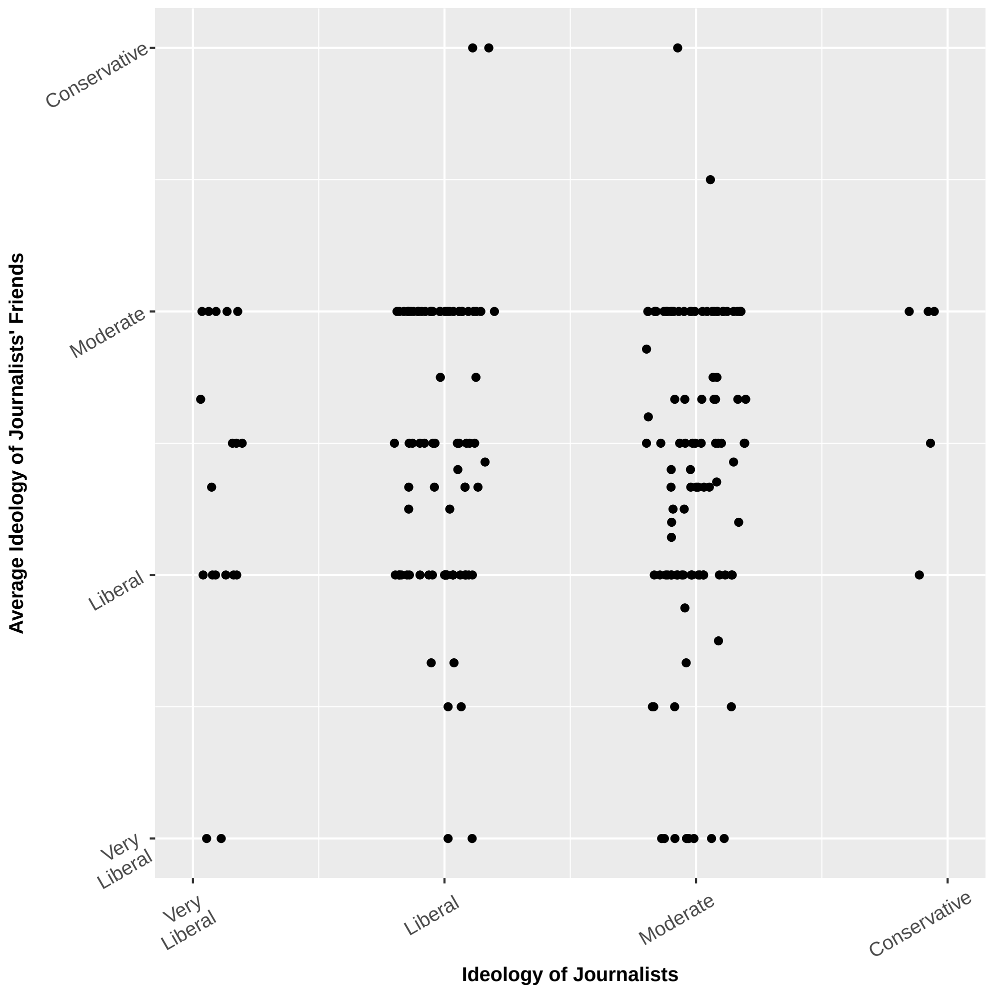


**Figure A.** The above figure shows the ideology scores of journalists and their friends. The horizontal axis represents journalists' political ideology. The vertical axis shows the average ideology score of each journalist's friends. Due to few observed cases of very conservative journalists, the figure omits these observations.

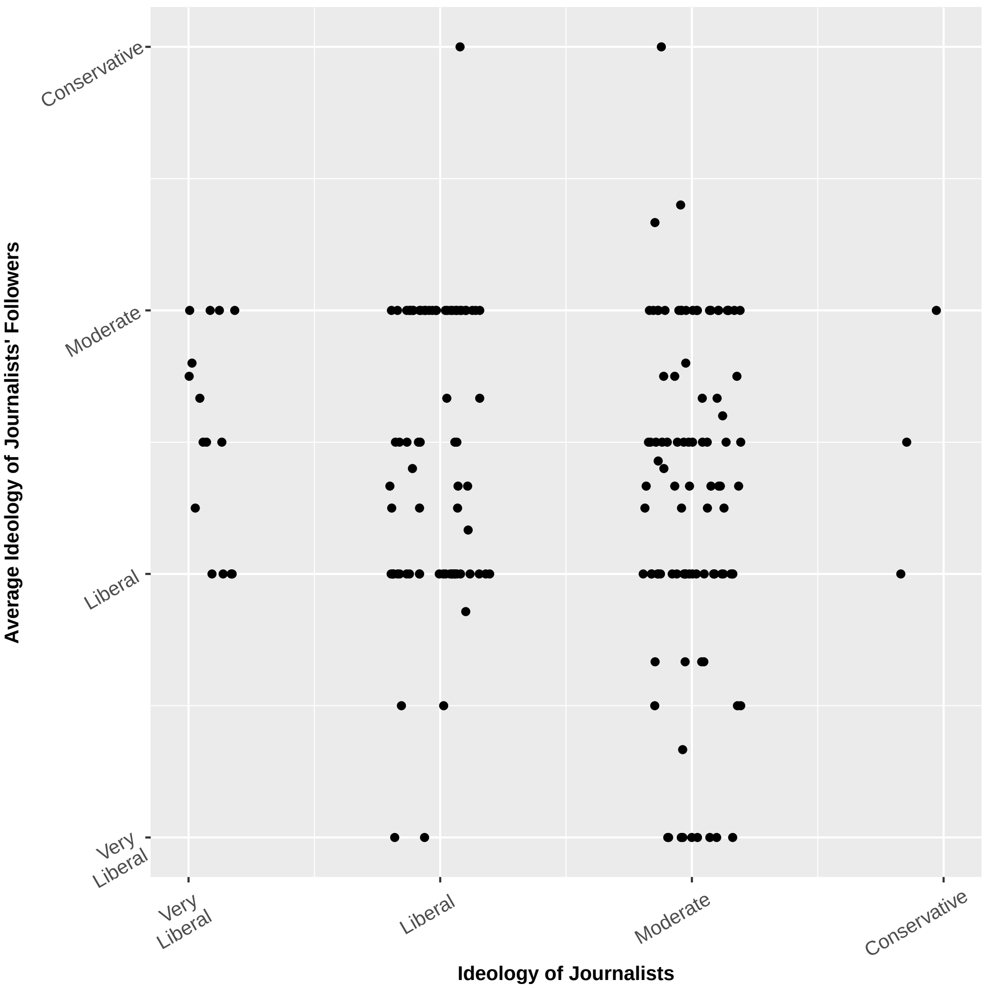


**Figure B.** The above figure shows the ideology scores of journalists and their followers. The horizontal axis represents journalists' political ideology. The vertical axis shows the average ideology score of each journalist's followers. Due to few observed cases of very conservative journalists, the figure omits these observations.
